# Endoscopic enucleation vs endoscopic vaporization procedures for benign prostatic hyperplasia: how should we choose: A protocol for systematic review and meta-analysis

**DOI:** 10.1097/MD.0000000000022882

**Published:** 2020-11-13

**Authors:** Xinbao Yin, Jun Chen, Hui Sun, Ming Liu, Zehua Wang, Benkang Shi, Xueping Zheng

**Affiliations:** a Department of Urology, Qilu Hospital of Shandong University; b Department of Geriatrics, The Affiliated Hospital of Qingdao University, Qingdao, Shandong, China.

**Keywords:** BPH, enucleation, lower urinary tract symptoms, vaporization

## Abstract

**Objective:**

To assess the safety and efficacy of different endoscopic procedures of the prostate techniques, by comparing endoscopic enucleation (EEP) and endoscopic vaporization procedures (EVP) of the prostate; and laser enucleation procedures (L-EEP) vs laser vaporization procedures (L-EVP) surgeries for benign prostatic hyperplasia.

**Methods:**

A systematic literature review was performed in December 2019 using PubMed, Embase and the Cochrane Library to identify relevant studies. Two analyses were carried out: (1) EEP vs EVP; and (2) L-EEP vs L-EVP. Efficacy and safety were evaluated using perioperative data, functional outcomes, including maximum urinary flow rate (Qmax), quality of life (QoL), international prostate symptom score (IPSS), postvoiding residual urine volume (PRV), and rate of complications. Meta-analyses were conducted using RevMan5.3.

**Results:**

Sixteen studies (4907 patients) evaluated EEP vs EVP, and 12 of them (4392 patients) evaluated L-EEP vs L-EVP. EEP showed improved functional outcomes compared with EVP. EEP was always presented a better Qmax at various follow-up times. EEP also associated with a reduced PRV and IPSS at 12 months postsurgery, an increased Qmax, and reduced IPSS and QoL score at both 24 and 36 months postsurgery. In addition, EEP was associated with less total energy utilized and retreatment for residual adenoma, but a longer catheterization time. Among other outcomes, there was no significant difference. L-EEP favors total energy used, retreatment for residual adenoma, and functional outcomes. L-EEP was associated with reduced PRV at 1, 6, and 12 months postsurgery, a greater Qmax at 6 and 12 months postsurgery, a lower IPSS at 12 months postsurgery, and higher Qmax and lower IPSS and QoL scores at 24 and 36 months postsurgery. However, there was no difference at 3 months postsurgery. No significant differences were observed for other perioperative data and complications.

**Conclusions:**

Both EEP and EVP displayed sufficient efficacy and safety for treating benign prostatic hyperplasia. EEP and L-EEP were favored in perioperative data, rate of complications, and functional outcomes. However, the clinical significance of those statistical differences was unclear. Hence, higher-quality randomized controlled trials may be needed to provide a clear algorithm.

## Abstract 2

Supplemental Digital Content is available in the text

## 1 Introduction

Benign prostatic hyperplasia (BPH) is ubiquitous in the aging male with prevalence increasing with age^[1]^ and affecting 50% of those older than 50 and 80% of those older than 80.^[2]^ Elderly males are frequently complaining of lower urinary tract symptoms (LUTS) which are due to bladder outflow obstruction secondary to BPH.^[3]^ There are many long-term complications due to untreated bladder outflow obstruction, such as detrusor failure, renal failure, recurrent urinary tract infections (UTIs), urinary retention, bladder diverticula, and bladder stones.^[4]^ If prostate tissue was removed in men with suspected BPH, symptoms and obstruction are reduced and resolved substantially.

Transurethral resection of the prostate (TURP) has been considered the gold standard surgical option for patients with moderate to severe LUTS secondary to BPH, especially for small/medium prostates.^[5,6]^ Substantial improvements on outcomes, including maximum urinary flow rate (Qmax), quality of life (QoL), international prostate symptom score (IPSS), and postvoiding residual urine volume (PRV) have added to its success. However, despite several technical and procedural improvements, TURP is still a potentially dangerous procedure, particularly in patients with larger prostates, indwelling catheters, bleeding disorders, or in patients undergoing anticoagulation therapy. Therefore, many endoscopic procedures and surgical skills using different energy sources, such as plasmakinetic TURP, plasmakinetic transurethral enucleation of the prostate, holmium laser enucleation of the prostate (HoLEP), and green light laser vaporization or enucleation of the prostate (green laser enucleation of the prostate), have been proposed to replace TURP as the new operative standard. These procedures have a satisfactory evidence base showing an advantage over TURP and a rise in use. Regardless of which kind of energy source is used, each approach of the transurethral procedure can be subdivided into three principles: resection, vaporization, and enucleation.^[7]^

So far, numerous studies have summarized the growing evidence supporting the use of these new techniques. In the present study, our aim was to perform a systematic review and meta-analysis using data from previously published studies to review the contemporary status of endoscopic enucleation (EEP) and the endoscopic vaporization procedures (EVP) techniques for the treatment of BPH. Moreover, we compared the safety and efficacy of the techniques that take advantage of the laser as energy sources, including laser enucleation procedures (L-EEP) and laser vaporization procedures (L-EVP).

## 2 Methods

### 2.1 Study criteria and search strategy

In our present systematic review and meta-analysis, we included the publications focus on patients treated surgically for symptomatic LUTS utilizing EEP and EVP. Studies using any kind of instrument for EEP and EVP were included, for example, holmium laser, green light laser, bipolar plasma, thulium laser, and transurethral vaporization in saline. When comparing L-EEP and L-EVP, only the holmium laser, greenlight laser, and thulium laser were included. The language was restricted to English. Studies were selected by searching PubMed, EMBASE, and the Cochrane Library up to December 2019. The search keywords included, but were not limited to, holmium laser enucleation of the prostate, HoLEP, transurethral enucleation, EEP, PVP, photoselective vaporization of the prostate, transurethral vaporization, endoscopic vaporization, GreenLight, transurethral prostatectomy, minimally invasive prostatectomy. We modified the search strategy as required for each electronic database. The bibliographies of included studies and recent reviews were hand-searched.

### 2.2 Selection of studies, data extraction, and methodological quality assessment

Studies that meet the prespecified inclusion criteria were selected. Abstracts of the identified articles were subjected to independent review by 2 authors. The full-text articles were retrieved for those studies that appeared to meet the inclusion criteria. Two reviewers independently extracted the data. To obtain missing data, the authors of the study were contacted. The methodological quality of randomized controlled trials (RCTs) was assessed according to the Jadad scale and not a RCT according to the Newcastle-Ottawa Scale (NOS) scale.

### 2.3 Outcome measures

The outcomes assessed included perioperative outcomes, complications, and efficacy of the surgery. The perioperative outcomes were assessed for a decrease in sodium, a drop in hemoglobin (HB) levels, irrigation length, hospital stay, conversion of surgical techniques, the total energy used, operative time, and catheterization time. Complications included capsular perforation, haematuria, clot retention, urge incontinence, stress incontinence, retreatment for residual adenoma, UTI, bladder neck contracture (BNC), urethral stricture, transient incontinence, blood transfusions, and urinary incontinence (including urge incontinence, stress incontinence, transient incontinence, and other incontinence not classified). For the efficacy of surgery, the following outcomes were used: maximum flow rates (Qmax), IPSS, QoL, and postvoid residual volume (PRV) at 1, 3, 6, 12, 24, and 36 months postsurgery.

### 2.4 Statistical analysis

RevMan5.3 was used to perform statistical analysis. Meta-analysis was conducted to generate summary statistics where possible. The weighted mean difference or standardized mean difference were calculated for continuous outcomes along with the 95% confidence interval (CI) and *P* value. Summary odds ratios (OR) and its 95% CI were calculated for binary outcomes. Statistical significance was defined as *P* < .05. For articles offering continuous data as median and interquartile range, we calculated the mean and standard deviation using the procedure described by Luo et al^[8]^ and Wan et al.^[9]^ The pooled results were calculated by the fixed-effect model [*I*^2^ (inconsistency) ≤ 50% and *P* ≥ .1]. Otherwise, the random effect model was used. Moreover, the effects of pooled results were determined by the *z* test, and *P* < .05 was considered statistically significant. Due to inconsistent data reporting, the meta-analysis was not feasible for all studies.

## 3 Results

### 3.1 Characteristics of included studies

**Figure 1:**
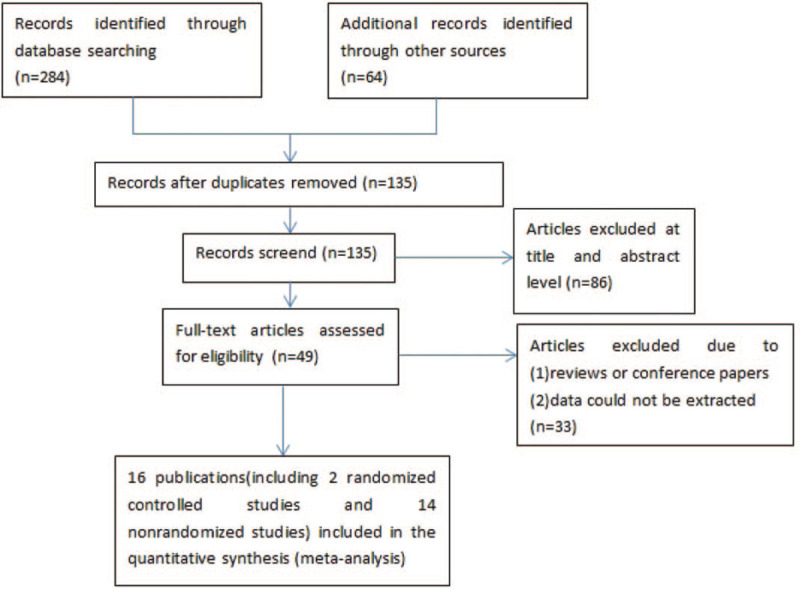
Flow diagram.

This systematic review and meta-analysis included 16 studies,^[10–25]^ with a total of 4907 patients (Fig. 1). The characteristics of the included studies are summarized in Table 1. In addition, Table 2 shows the baseline demographic and clinical characteristics of each study. All trials were published in English. The median sample size was 192.5 patients (range: 26–1184). The follow-up time ranged from 12 months to 60 months. Among the enrolled studies, 12 studies^[10–13,15–18,20,22,23,25]^ (4392 patients) compared L-EEP and L-EVP.

**Table 1:** A summary of comparative studies.

Year, author	Country	Study period	Study design	Surgical skills	Cases, n.	Inclusion criteria	Follow-up, mo	Study quality
2012, Elhilali et al^[10]^	Egypt	October 2008 to October 2010	RCT	HoLEP/ PVP	43/37	IPSS > 9, PV >60 ml, Qmax <15 ml/s	12	1^∗^
2013, Elterman et al^[11]^	USA	September 2001 to May 2009	Retrospective case-control	GreenLEP/PVP	170/97	IPSS > 8, Qmax <10 ml/s	36	7^†^
2015, Jaeger et al^[12]^	USA	2009 to 2012	Retrospective case-control	HoLEP/PVP	72/31	PVR of >300 mL	–	7^†^
2015, Cho et al^[13]^	Korea	2005 to 2011	Retrospective case-control	HoLEP/PVP	273/213	–	12	7^†^
2015, Geavlete et al^[14]^	Romania	January 2009 to May 2013	RCT	BPEP/TUVis	80/80	IPSS > 19, PV > 80 ml, Qmax <10 ml/s	12	2^∗^
2015, Elkoushy et al^[15]^	Egypt	March 1998 to July 2014	Retrospective case-control	HoLEP/PVP	809/291	–	12	8^†^
2016, Misrai et al^[16]^	France	April 2011 to March 2014	Retrospective case-control	GreenLEP/PVP	60/60	PV > 80 ml	12	7^†^
2016, Kim et al^[17]^	Korea	April 2011 to March 2014	Retrospective case-control	HoLEP/PVP	162/176	IPSS > 7, PV < 40 ml,Qmax < 15 ml/s, PVR > 100 ml	12	7^†^
2017, Cindolo et al^[18]^	Italy	July 2012 to November 2015	Retrospective case-control	GreenLEP/ PVP	35/139	–	–	7^†^
2017, Mu et al^[19]^	China	February 2011 to December 2013	Retrospective case-control	BTUEP/PVP	39/42	IPSS > 12, PV > 70 ml, Qmax <15 ml/s	12	9^†^
2017, Yoo et al^[20]^	Korea	January 2008 to January 2017	Retrospective case-control	GreenLEP/ PVP	199/64	PV ≥ 40 ml	12	7^†^
2017, Wang et al^[21]^	China	February 2011 to July 2012	Retrospective case-control	PKEP/PVP	101/110	IPSS > 12,Qmax <15 ml/s	12	7^†^
2018, Castellan et al^[21]^	Italy	2014 to 2017	Retrospective case-control	ThuVEP/PVP	158/93	–	12	7^†^
2019, Sun et al^[23]^	Korea	January 2008 to March 2014	Retrospective case-control	HoLEP/PVP	745/439	–	60	8^†^
2019, Kim et al^[24]^	Korea	January 2017 to June 2018	Retrospective case-control	HoLEP/BPVP	32/31	IPSS > 7, PV < 40 ml,Qmax <15 ml/s, PRV > 100 ml	–	7^†^
2019, Prudhomme et al^[25]^	France	January 1, 2013 to April 30, 2018	Retrospective case-control	HoLEP/PVP	17/9	IPSS > 10, Qmax<10 mL/s, PVR >100 mL	12	7^†^

**Table 2:** Baseline demographic and clinical characteristics.

	Age (yrs)		PV (ml)		PSA (ng/ml)		Qmax (ml/s)		PVR (ml)		IPSS		QoL	
Year, author	EEP	EVP	EEP	EVP	EEP	EVP	EEP	EVP	EEP	EVP	EEP	EVP	EEP	EVP
2012, Elhilali et al^[10]^	71.5 ± 7.7	73.2 ± 8.5	91.3 ± 23.2	89. ± 16.6	5.4 ± 3.4	7.5 ± 6.5	8.1 ± 2.7	8.9 ± 2.1	268 ± 237	272 ± 285	22.4 ± 4.6	21.8 ± 4.7	4.2 ± 1.3	4.2 ± 1.2
2013, Elterman et al^[11]^^†^	70.0	69.1	83	63	2.2	2.1	9.9	9.8	116	75	16.7	16.7	4	5
2015, Jaeger et al^[12]^^‡^	71 (62–77.75)	70 (62–76)	88.5 (57–126)	49 (31–75)	4.5 (3.1–8.6)	2.4 (0.8–4.5)	5.1 (3–8.3)	5.6 (3.9–9.9)	555 (390–700)	473 (327–628)	18^∗^ (12.5–23)	21^∗^ (15.5–25.5)	–	–
2015, Cho et al^[13]^	68.9 ± 6.4	67.7 ± 6.7	55.1 ± 20.9	50.6 ± 22.0	3.2 ± 3.6	2.9 ± 3.3	10.6 ± 3.6	11.6 ± 3.2	78.1 ± 104.9	72.0 ± 70.5	18.3 ± 7.1	19.0 ± 7.4	4.1 ± 1.1	4.2 ± 1.2
2015, Geavlete et al^[14]^	68.5 ± 8.5	67.5 ± 9.1	122.6 ± 30.7	126.7 ± 33.1	8.16 ± 3.43	8.03 ± 3.54	6.6 ± 1.6	6.9 ± 1.8	134.1 ± 86.8	158.3 ± 114.9	24.7 ± 3.3	24.4 ± 3.1	4.1 ± 1.1	4.3 ± 1.0
2015, Elkoushy et al^[15]^	71.9 ± 8.1	71.2 ± 9.0	94.9 ± 50.3	49.9 ± 25.3	5.79 ± 10.1	3.22 ± 9.5	7.57 ± 3.63	8.20 ± 3.35	181.6 ± 187.1	150.2 ± 156.5	19.6 ± 6.8	18.3 ± 7.7	3.52 ± 1.44	3.66 ± 1.35
2016, Misrai et al^[16]^^‡^	68 (63–74)	70 (65–77)	100 (80–120)	100 (85–110)	4.3 (2.8–7.5)	4.3 (2.8–7.5)	5.2 (4.0- 6.0)	6.5 (4.9–8.0)	50 (0–150)	100 (100- 200)	17 (14–20)	18 (14–21)	5.2 (4.0–6.0)	6.5 (4.9–8.0)
2016, Kim et al^[17]^	69.5 ± 7.4	70.7 ± 8.1	29.2 ± 6.7	30.2 ± 6.1	1.9 ± 1.9	2.0 ± 1.8	9.3 ± 4.3	8.7 ± 4.9	86.8 ± 116.3	133.0 ± 115.7	21.5 ± 8.5	20.4 ± 7.8	4.2 ± 1.1	4.1 ± 2.2
2017, Cindolo et al^[18]^	68.2 ± 7.4	69.3 ± 9.0	90 (75–125)^‡^	60 (45–88) ^‡^	4.3 (3.7–6.8)^‡^	2.3 (1.3–3.8)^‡^	8 (6–9)^‡^	9 (8–11)^‡^	–	–	21 (18–24)^‡^	25 (23–27)^‡^	–	–
2017, Mu et al^[19]^	71.15 ± 6.33	70.45 ± 5.52	88.32 ± 21.28	83.07 ± 10.90	6.01 ± 3.43	5.55 ± 2.72	5.75 ± 2.76	5.73 ± 3.33	123.18 ± 103.63	135.86 ± 117.02	22.87 ± 5.00	21.60 ± 5.12	4.55 ± 0.90	4.68 ± 0.88
2017, Yoo et al^[20]^	69.4 ± 9.3	68.7 ± 6.2	74.3 ± 33.1	63.5 ± 21.1	5.1 ± 4.2	4.6 ± 3.7	9.1 ± 5.4	8.8 ± 3.2	90.6 ± 118.8	101.1 ± 148.8	20.4 ± 8.7	19.8 ± 9.3	4.2 ± 1.4	4.1 ± 1.4
2017, Wang et al^[21]^	69.51 ± 7.34	68.87 ± 6.40	62.97 ± 14.19	61.98 ± 11.98	3.04 ± 2.79	3.23 ± 3.26	6.60 ± 2.35	6.79 ± 2.54	92.49 ± 30.49	94.25 ± 33.76	22.37 ± 5.06	21.74 ± 4.74	4.59 ± 0.95	4.62 ± 0.89
2018, Castellan et al^[22]^	70.0 ± 8.5	68.9 ± 8.5	52.5 (26)	58 (25)	2.7 (2)	2.7 (2.9)	8.4 ± 2.9	8.0 ± 2.8	–	–	25.5 (4)	25 (9)	–	–
2019, Sun et al^[23]^	68.7 ± 6.6	68.0 ± 8.0	60.0 ± 25.5	51.4 ± 32.0	3.6 ± 4.1	5.2 ± 8.0	10.3 ± 4.6	10.6 ± 5.5	67.2 ± 92.3	83.2 ± 117.3	19.2 ± 7.4	20.6 ± 8.1	4.2 ± 1.0	4.3 ± 1.0
2019, Kim et al^[24]^	70.23 _ 5.61	69.81 _ 7.54	36.01 _ 25.78	37.04 _ 29.41	2.18 _ 6.13	2.11 _ 6.38	9.45 _ 3.59	9.09 _ 2.37	155.7 _ 104.9	149.8 _ 68.1	21.8 _ 8.5	20.7 _ 8.1	9.09 _ 2.37	9.45 _ 3.59
2019, Prudhomme et al^[25]^^‡^	67.1 (63.9–71.7)	65.6 (58.9–74.1)	56.0 (50.0–70.0)	40.0 (40.0–40.0)	2.7 (1.7–8.4)	19.5 (12.0–27.0)	10.5 (6.0–14.0)	8.6 (5.0–15.0)	–	–	14.0 (6.5–28.5)	16.0 (15.0–16.0)	–	–

### 3.2 Perioperative outcomes in EEP vs EVP

**Figure 2:**
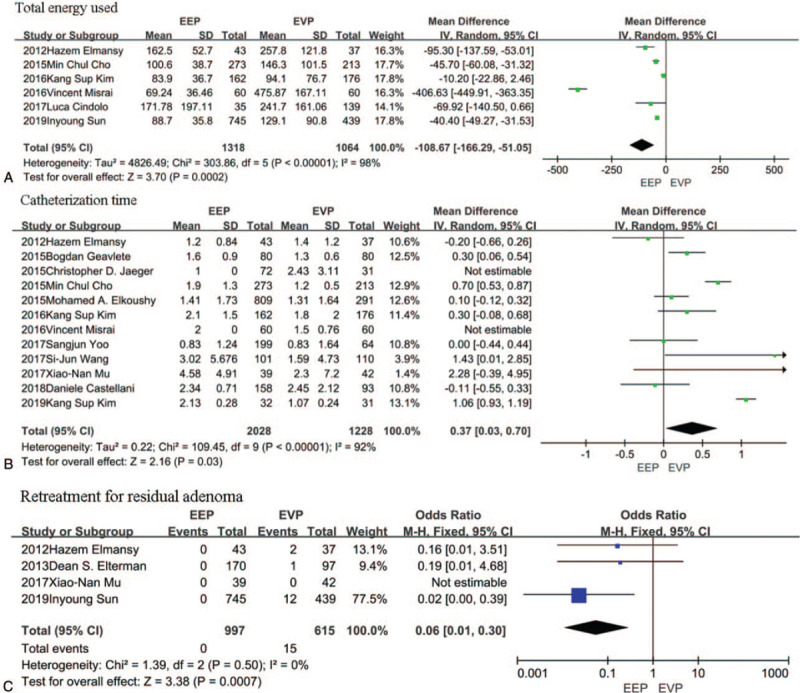
A-B, The forest plot of pooled estimates of perioperative outcomes in EEP vs EVP. C, The forest plot of pooled estimates of postoperative complications in EEP vs EVP. EEP = endoscopic enucleation, EVP = endoscopic vaporization procedures.

For all included studies, the total energy used was lower in the EEP group by 108.67 minutes compared with the EVP group (95% CI, [−166.29, −51.05]; *P* = .0002); However, catheterization time was shorter in the EVP group by 0.37 days (95% CI, [0.03,0.70]; *P* = .03) (Fig. 2A-B)

### 3.3 Postoperative complications in EEP vs EVP

In the EEP group, there was no incidence of retreatment for residual adenoma, However, 15 patients required retreatment for residual adenoma in the EVP group (OR: 0.06; 95% CI, [0.01,0.53]; *P* = .0007) (Fig. 2C). No differences were noted in capsular perforation, haematuria, clot retention, urge incontinence, stress urinary incontinence, UTI, BNC, urethral stricture, transient incontinence, blood transfusions, and urinary incontinence. (see Supplementary information).

### 3.4 Efficacy of operation in EEP vs EVP

#### 3.4.1 One month postsurgery

**Figure 3:**
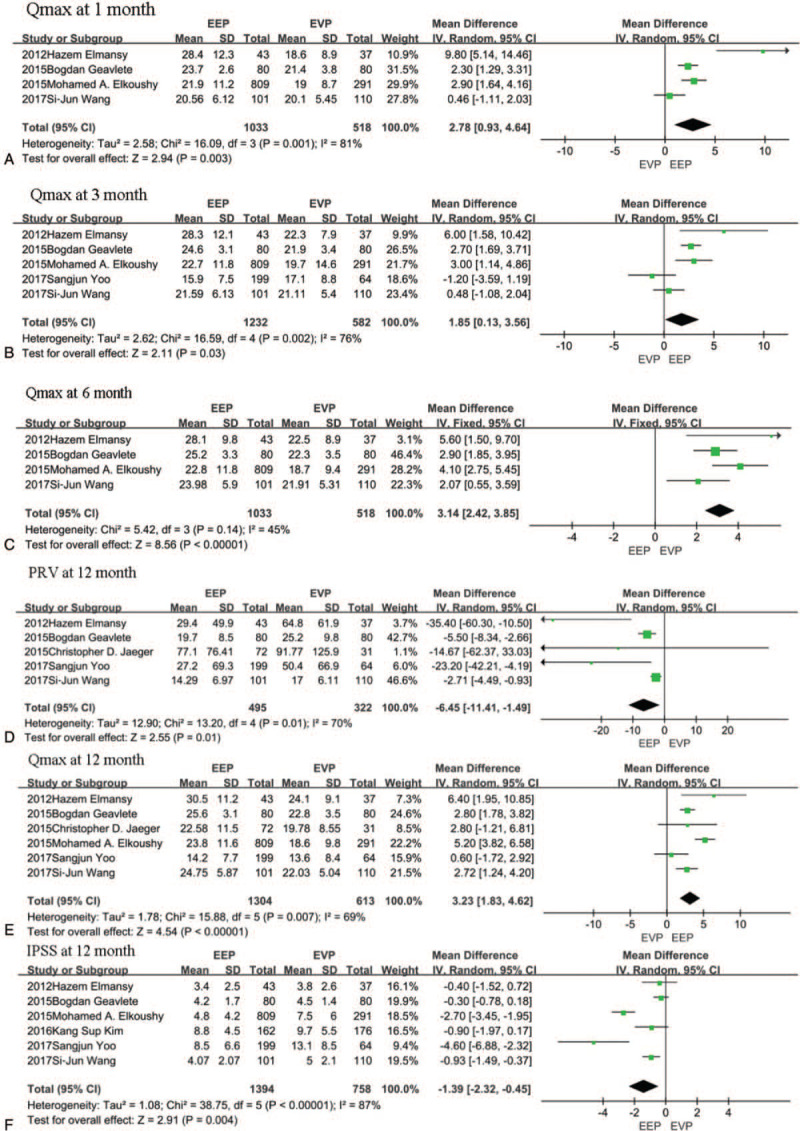
A, The forest plot of pooled estimates of the efficacy of operation at 1 month of postoperation in EEP vs EVP. B, The forest plot of pooled estimates of the efficacy of operation at 3 months of postoperation in EEP vs EVP. C, The forest plot of pooled estimates of the efficacy of operation at 6 months of postoperation in EEP vs EVP. D–F, The forest plot of pooled estimates of efficacy of operation at 12 months of postoperation in EEP vs EVP. EEP = endoscopic enucleation, EVP = endoscopic vaporization procedures

At 1 month postsurgery, IPSS, and QoL data were obtained from 7 studies.^[10,14,15,17,20,21,24]^ PRVand Qmax were compared in 3^[10,14,21]^ and 4 studies,^[10,14,15,21]^ respectively. EEP presented a better Qmax than EVP at 1 month postsurgery (2.78 ml/s, 95% CI [0.93, 4.64], *P* = .003) (Fig. 3A). However, there were no significant differences in IPSS, PRV, and QoL. (see Supplementary information).

#### 3.4.2 Three months postsurgery

The studies that reported the efficacy of surgery differed in IPSS, QoL, PRV, and Qmax. We acquired the IPSS data from 6 studies.^[10,14,15,17,21,24]^ The studies that reported QoL at the 1 month postsurgery also reported QoL at 3 months postsurgery.^[10,14,15,17,20,21,24]^ PRV was compared in four studies^[10,14,20,21]^ and Qmax in 5 studies.^[10,12,14,15,20,21]^ EEP showed significant benefits in terms of Qmax in the third month postsurgery too, (1.85 ml/s, 95% CI [0.13, 3.56], *P* = .03) but no significant differences were observed in terms of IPSS, PRV, and QoL (Fig. 3B).

#### 3.4.3 Six month postsurgery

Data for IPSS and QoL were obtained from 6 studies at 6 months postsurgery.^[10,14,15,17,21,24]^ The same studies reported PRVat 1 month^[10,14,21]^ and QoL^[10,14,15,21]^ at 3 months. Only Qmax was significantly different between EEP and EVP. EEP was associated with a greater Qmax (3.14 ml/s, 95% CI [2.42, 3.85], *P* < .00001) in the pooled data analysis (Fig. 3C).

#### 3.4.4 Twelve months postsurgery

Six studies^[10,14,15,17,20,21]^ reported the IPSS and QoL. Five studies^[10,12,14,20,21]^ and 6 studies^[10,12,14,15,20,21]^ compared PRV and Qmax respectively. Our meta- analysis showed no significant difference in QoL between EEP and EVP. EEP procedures, however, appeared to be associated with a higher Qmax (3.23 ml/s, 95% CI [1.83, 4.62], *P* < .00001), less PRV (6.45 ml, 95% CI [−11.41, −1.49], *P* = .01) and lower IPSS score (−1.39, 95% CI [−2.32, −0.45], *P* = .004) (Fig. 3D–F).

#### 3.4.5 Twenty-four and 36 months postsurgery

**Figure 4:**
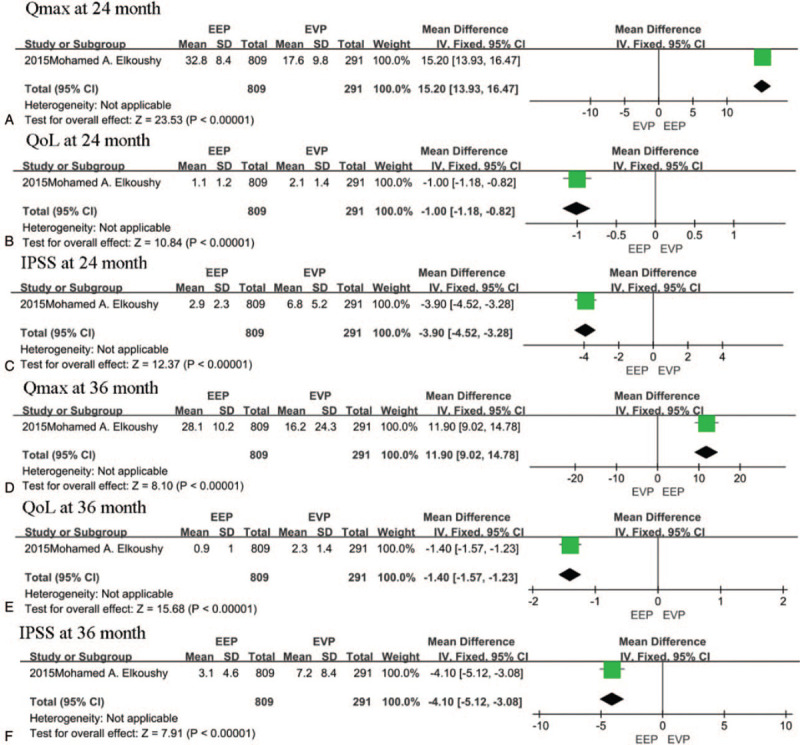
A–F, The forest plot of pooled estimates of the efficacy of operation at 24 and 36 months of postoperation in endoscopic enucleation vs endoscopic vaporization procedures.

Only the study by Elkoushy et al^[15]^ compared IPSS, QoL, and Qmax between EEP and EVP. The pooled analysis showed that EEP techniques were associated with a higher Qmax (15.20 ml/s, 95% CI [13.93, 16.47], *P* < .00001 at 24 month after surgery and 11.90 ml/s, 95% CI [9.02, 14.78], *P* < .00001 at 36 month after surgery), lower IPSS (−3.90, 95% CI [−4.52, −3.28], *P* < .00001 at 24 month after surgery and −4.10, 95% CI [−5.12, −3.08], *P* < .00001 at 36 month after surgery)and QoL score (−1.00, 95% CI [−1.18, −0.82], *P* < .00001 at 24 month after surgery and −1.40, 95% CI [−1.57, −1.23], *P* < .00001 at 36 month after surgery) at both follow-up times (Fig. 4A–F).

### 3.5 Perioperative outcomes in L-EEP vs L-EVP

**Figure 5:**
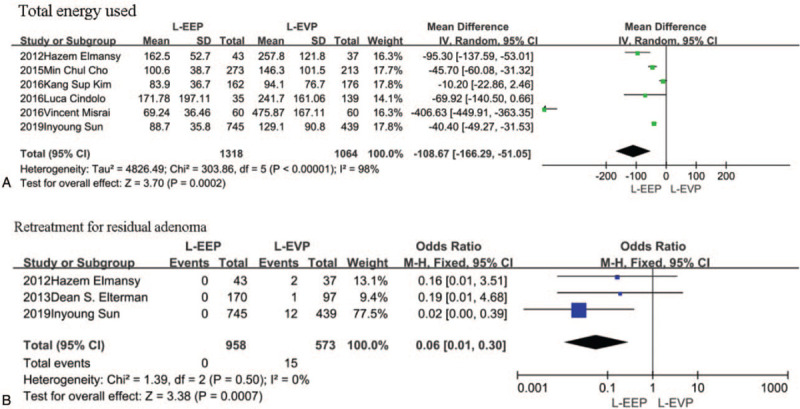
A, The forest plot of pooled estimates of the efficacy of perioperative outcomes in L-EEP vs L-EVP. B, The forest plot of pooled estimates of postoperative complications in L-EEP vs L-EVP. L-EEP = laser enucleation procedures, L-EVP = laser vaporization procedures.

Similar to the results of perioperative outcomes in EEP vs EVP, the total energy used was the only statistically different pooled data in our analysis, L-EEP procedures using less energy in operation (108.67 minutes 95% CI, [−166.29, −51.05]; *P* = .0002) (Fig. 5A). However, no statistical differences were observed between L-EEP and L-EVP in terms of HB level drop, hospital stay, conversion of surgical techniques, operative time, and catheterization time in the pooled data. There were insufficient data to analyze the decrease in sodium, or irrigation length during perioperative periods. (see Supplementary information).

### 3.6 Postoperative complications in L-EEP vs L-EVP

In the L-EEP group, the rate of retreatment for residual adenoma was lower than in the L-EVP group (OR: 0.06; 95% CI, [0.01,0.30]; *P* = .0007) (Fig. 5B). No differences were noted in capsular perforation, haematuria, clot retention, urge incontinence, stress urinary incontinence, retreatment for residual adenoma, UTI, BNC, urethral stricture, transient incontinence, blood transfusions, and urinary incontinence. (see Supplementary information).

### 3.7 Efficacy of operation in L-EEP vs L-EVP

#### 3.7.1 One month postsurgery

**Figure 6:**
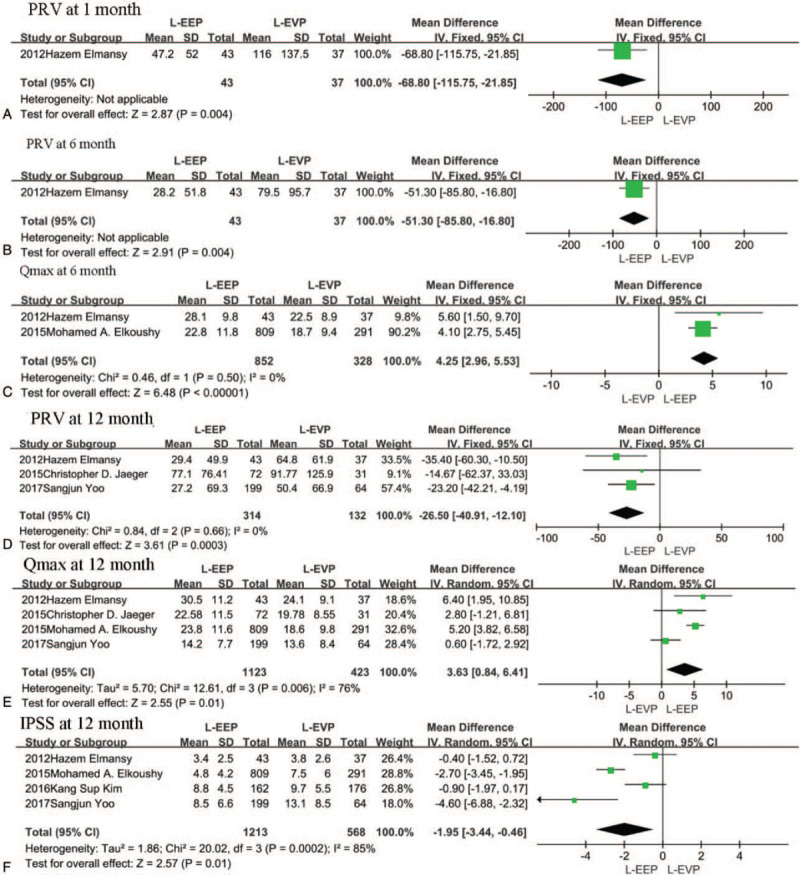
A, The forest plot of pooled estimates of the efficacy of operation at 1 month of postoperation in L-EEP vs L-EVP. B-C, The forest plot of pooled estimates of the efficacy of operation at 6 months of postoperation in L-EEP vs L-EVP. D–F The forest plot of pooled estimates of efficacy of operation at 12 months of postoperation in L-EEP vs L-EVP. L-EEP = laser enucleation procedures, L-EVP = laser vaporization procedures.

Only Elmansy et al^[10]^ compared PRV between the L-EEP group and the L-EVP group. The pooled data showed less PRV in the L-EEP group at 1 month after surgery (68.8 ml, 95% CI [−115.75, −21.85], *P* = .004) (Fig. 6A). However, no significant differences were noted in IPSS and QoL from 4 studies^[10,15,17,20]^ and Qmax from 2 studies.^[10,15]^ (see Supplementary information).

#### 3.7.2 Three month postsurgery

This study showed no statistical differences in both groups regarding IPSS,^[10,15,17,20]^ Qmax,^[10,15,20]^ QoL,^[10,15,17,20]^ and PVR^[10,20]^ at 3 months in the pooled data analysis. (see Supplementary information).

#### 3.7.3 Six month postsurgery

In the pooled data from three studies with 1581 patients, IPSS and QoL showed no statistical differences between L-EEP and L-EVP 6 months postsurgery.^[10,15,17]^ However, L-EEP was associated with a greater Qmax (4.25 ml/s, 95% CI [2.96, 5.53], *P* < .001)^[10,15]^ and less PRV (51.3 ml, 95% CI [−85.8, −16.8], *P* = .004)^[10]^ in the pooled data analysis (Fig. 6B-C).

#### 3.7.4 Twelve of months postsurgery

In L-EEP group at 12 months post-surgery, PRV(26.5 ml, 95% CI [−40.91, −12.1], *P* = .0003),^[10,12,20]^ Qmax (3.63 ml/s, 95% CI [0.84, 6.41], *P* = .01)^[10,12,15,20]^ and IPSS (1.95, 95% CI [−3.44, −0.46], *P* = .01)^[10,15,17,20]^ (Fig. 6D–F) were better compared with the L-EVP group. However, there was no significant difference between L-EEP and L-EVP for the QoL score of the pooled data.^[10,15,17,20]^ (see Supplementary information).

#### 3.7.5 Twenty-four and 36 months postsurgery

**Figure 7:**
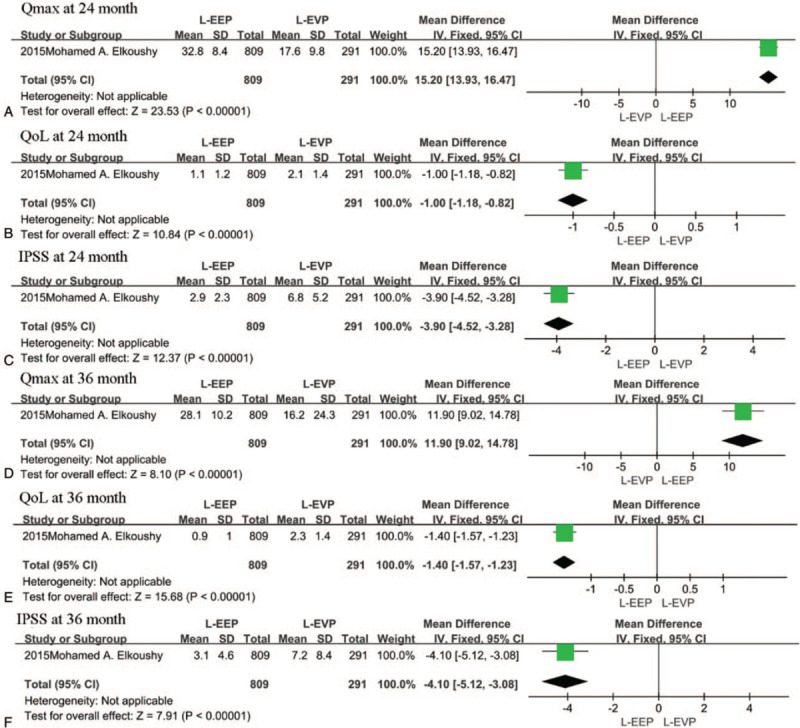
A–F The forest plot of pooled estimates of the efficacy of operation at 24 and 36 months of postoperation in laser enucleation procedures vs laser vaporization procedures.

Only the study by Elkoushy et al^[15]^ compared IPSS, QoL, and Qmax between EEP and EVP at 24- and 36 months postsurgery. The pooled analysis showed that EEP techniques were associated with a higher Qmax (15.20 ml/s, 95% CI [13.93, 16.47], *P* < .00001 at 24 month after surgery and 11.90 ml/s, 95% CI [9.02, 14.78], *P* < .00001 at 36 month after surgery), lower IPSS (−3.90, 95% CI [−4.52, −3.28], *P* < .00001 at 24 month after surgery and −4.10, 95% CI [−5.12, −3.08], *P* < .00001 at 36 month after surgery) and QoL score (−1.00, 95% CI [−1.18, −0.82], *P* < .00001 at 24 month after surgery and −1.40, 95% CI [−1.57, −1.23], *P* < .00001 at 36 month after surgery) at both follow-up times (Fig. 7A–F).

## 4 Discussion

For many years, TURP is still considered gold standard for the small/medium prostates and open prostatectomy^[26]^ was the most appropriate choice for men with large gland volumes. However, TURP is still accompanied by some life-threatening risks, such as a 2% to 4.8% rate of hemorrhage requiring blood transfusion,^[27]^ and elevated morbidity occurred in open prostatectomy.^[28]^ Therefore, diverse transurethral techniques have been adopted in recent years which possess excellent functional outcomes and lower complication rates. Among these new procedures, EEP and EVP seem to be the methods with the most potential since high-quality studies give a demonstration of persistent improvement in the QoL and other functional outcomes, as well as a favorable safety profile.^[1,7,29–31]^

Some superb meta-analyses have been published comparing diverse endoscopic procedures^[31]^ and head-to-head compared specific technologies, such as HoLEP vs bipolar transurethral resection of the prostate,^[32]^ thulium laser enucleation of the prostate vs TURP.^[33]^ Although EEP is a widely used surgical procedure, it has never been investigated as a whole and compared with EVP techniques. It is difficult but important to select the best surgical treatment for BPH. Hence, it is worth comparing different surgical procedures, whether it is through direct or indirect studies. As far as we know, our meta-analysis represents the first study to indirectly compare EEP with EVP in terms of the efficacy and safety for the treatment of BPH. Moreover, since laser surgery is a popular procedure today but was rarely used in the last century,^[34]^ we further analyzed the differences between L-EEP and L-EVP.

During the entire postoperative follow-up, greater Qmax values were obtained with enucleation methods, including bipolar plasma enucleation of the prostate, plasmakinetic transurethral enucleation, HoLEP, thulium enucleation of the prostate, and green laser enucleation of the prostate. even after 24 to 36 months. Enucleation methods were also associated with better PRV, IPSS, and QoL compared with vaporization methods when the postoperative follow-up period exceeded 12 months. However, when laser techniques were considered, better functional outcomes of Qmax only became apparent 6 months postsurgery. However, the lower PRV seemed to persist throughout the follow-up duration (except 3 months after operation) and the differences with IPSS and QoL could be observed over 12 months following surgical treatment.

EEP methods imitate open prostatectomy and remove more tissues using fibreoptic lasers or bipolar loops.^[35]^ Therefore, it is not surprising that enucleation methods yielded the greatest Qmax values compared with resection and vaporization methods, since more tissues were removed using enucleation methods than vaporization methods.^[36]^ In a systematic review and network meta-analysis performed by Huang et al,^[37]^ EEP procedures have been shown to improve Qmax by about 1.71 to 1.98 mL/s and 4.12 to 4.82 mL/s at 6 to 12 and 24 to 36 months postsurgery, respectively, compared with EVP procedures which have been considered clinically significant. In this study, EEP was observed to improve Qmax by 2.78 and 1.85 mL/s than vaporization methods at 1 month and 3 months, and by 3.14, 3.23, 15.2, and 11.9 mL/s more at 6, 12, 24, and 36 months following surgery, respectively. Accordingly, it was clinically significant for the difference of Qmax between EEP and EVP procedures.

As we compared the perioperative data of EEP and EVP, both showed effective outcomes. On the other hand, based on our meta-analysis, EVP favored significant differences with respect to catheterization time, while there was no significant difference between the L-EEP and L-EVP groups. Our analysis also showed that EVP and L-EVP methods seemed to generate higher total energy used during surgery compared with EEP and L-EEP. EEP is widely believed to increase the risk of urinary incontinence and capsular perforation.^[38]^ Nevertheless, our study did not observe any differences in the rate of implications between groups. A lower rate of retreatment for residual adenoma in EEP and L-EEP was observed. This could be explained by the fact that EEP methods remove whole hyperplastic adenoma of the prostate as open prostatectomy, while EVP methods remove less apical prostate tissue to prevent sphincter injury.^[39]^ Therefore, in order to overcome the shortcomings of vaporization, some surgeons resect the apex of the prostate after vaporization.^[40]^

A published network meta-analysis of 88 randomly controlled trials with 15 procedures found that HoLEP was the first choice for PRV values, although diode laser vaporization of prostate gave better results in terms of IPSS and Qmax.^[34]^ Another network meta-analysis comparing different lasers for surgical treatment of BPH, comprising 36 randomized studies involving 3831 patients found that HoLEP was the most advantageous operative procedure for improving PRV.^[41]^ Our results indicated that enucleation technology is more effective than vaporization, regardless of the laser energy that was utilized.

Huang et al^[37]^ reported superior functional outcomes and parallel safety when comparing enucleation methods to vaporization methods. Such results were confirmed by our meta-analysis again. Regarding laser energy, the results were similar when comparing L-EEP and L-EVP. However, Zhang et al^[41]^ comparing different lasers for the treatment of BPH found that dysuria was the most frequent short-term complication in patients treated with green laser vapo-enucleation of the prostate and HoLEP, but was rarely seen in Nd:YAG laser with vaporization. When long-term complications-related outcomes were considered, BNC or stenosis was oftentimes found in KTP/Nd:YAG with vaporization and HoLEP comparing with green laser vaporization of the prostate and diode laser with vaporization.

Our study included only 2 RCTs. Hence, we must interpret the results within the context of some limitations. First, the follow-up time varied from study to study: data for more than 12 months of follow-up were obtained from only 1 study,^[15]^ and the majority of the included studies had a maximum follow-up of up to 1 year.^[10,13,14,16,17,19–22,24,25]^ Consequently, we lacked the data to evaluate the differences in long-term efficacy and safety between EEP and EVP. Second, as a result of rare complications and zero events reported by some studies, the pooled ORs were less precise. Third, there existed high heterogeneity in most analyses, which could be interpreted as the difference in each trial studied, such as the initial volume of prostate, the degree of urodynamic obstruction, and the level of experience of the surgeons. Fourth, because of a lack of standardized definition and techniques, we did not distinguish vapo-enucleation from enucleation. Hence, further investigations are required to evaluate the differences in outcomes between vapo-enucleation and enucleation or vaporization methods. Nevertheless, this review still enjoys several advantages. To our knowledge, this study is the first comprehensive systematic review and meta-analysis that focuses on comparing EEP and EVP methods for treatment of BPH, and that offers an evaluation of their efficacy and safety, with a view of providing valuable insights and recommendations for clinician surgeons.

## 5 Conclusion

Our study showed that EEP and EVP provide efficacy and safety for the treatment of BPH. Most perioperative data existed no significant difference between both groups, but EEP favors total energy used and retreatment for residual adenoma and reduces catheterization time. EEP shows better functional outcomes than EVP. Compared with L-EVP, L-EEP provides greater total energy used and retreatment for residual adenoma with the same complications and better functional profiles. However, the clinical significance of these findings remains unclear. Therefore, more long-term, larger-scale, and well-designed head-to-head RCTs are needed to provide a clear direction as to which techniques to select in clinical conditions.

## Acknowledgments

We are grateful to the participants and researchers of the primary studies identified for this meta-analysis and the editors and the anonymous reviewers for the comments provided and the valuable inputs. We thank our institutions for allowing us to perform this study.

## Author contributions

**Data curation:** Jun Chen, Ming Liu.

**Formal analysis:** Hui Sun.

**Software:** Zehua Wang.

**Visualization:** Benkang Shi.

**Writing – original draft:** Xinbao Yin.

**Writing – review & editing:** Xueping Zheng.

## Supplementary Material

Supplemental Digital Content
